# Poisoning by Organophosphate Pesticides: A Case Report

**DOI:** 10.7759/cureus.29842

**Published:** 2022-10-02

**Authors:** Gudisa Bereda

**Affiliations:** 1 Department of Pharmacy, Negelle Health Science College, Negelle, ETH

**Keywords:** acetylcholinesterase enzyme, organophosphate pesticide poisoning, case report, atropine sulfate, an emergency department

## Abstract

In many parts of the world, particularly in impoverished nations like Ethiopia, organophosphate compounds operate as suicide agents, are frequently employed as pesticides, and are strong inhibitors of the acetylcholinesterase enzyme. A 21-year-old Ethiopian female, a university student, was admitted to an emergency department on June 22, 2022, with a two-hour history of nausea and elevated secretions of salivation via the mouth. She had no previous history of psychiatric or neurological disorders, but three days before her admission, she quarreled with her boyfriend, became extremely depressed, and decided to commit suicide. She had a two-hour history of nausea and intermittent vomiting and a one-hour history of persistent vomiting, increased salivation secretions through the mouth, chills, progressive sweating, difficulty breathing, and dizziness. Upon admission, her neurological examination in the emergency department revealed a Glasgow Coma Scale score of 9/15. On admission, she was placed on two liters per minute of intranasal oxygen via the nasal cannula. On the same day, she was given atropine 0.15 mg intravenously, and the dose was doubled every 10 minutes until atropinization was achieved, and a bolus dose of 500ml of 0.9% of normal saline was initiated immediately.

## Introduction

Adults are frequently poisoned via deliberate eating, inhalation, or contact with hazardous substances [1]. Insecticides made of organophosphate compounds are widely utilized worldwide, particularly in agricultural contexts [2]. Organophosphate chemicals are widely obtained over the counter, and little thought is paid to their management even though they have been used for suicide, especially in developing countries [3]. Organophosphate insecticides have dangerous side effects, including respiratory collapse, severe poisoning increased saliva and tear production, confusion, vomiting, sweating, small pupils with acute fasciculation, and immobility [4,5]. Organophosphate poisoning results in three primary syndromes: (i) acute cholinergic syndrome; (ii) intermediate syndrome; and (iii) delayed polyneuropathy [6]. Atropine, pralidoxime, and benzodiazepines like diazepam are the most frequently prescribed pharmaceutical treatments for organophosphate chemical poisoning [7]. The main treatment for organophosphate chemical poisoning is atropine, which is given intravenously to restore adequate cardiorespiratory function quickly [8]. This case report shows an adult girl who was poisoned by an organophosphate pesticide.

## Case presentation

On June 22, 2022, a 21-year-old Ethiopian female who was a university student was admitted to the emergency room with nausea that had been present for two hours and increased salivation. At 4:15 PM, she drank organophosphate insecticide. After two hours of drinking the organophosphate insecticide to try suicide, she was brought to the student clinic in the university at 6:35 PM with a history of semi-consciousness by students from her dorms. She had no prior history of neurological or psychiatric illnesses. She had never before been known to attempt suicide, but three days before her admission, she quarreled with her boyfriend, became extremely depressed, and decided to commit suicide. She was leaving a note on her bed that read, "I shall see you in paradise if you will join it." Since there are no restrictions on the open availability of organophosphate insecticides in Ethiopia, she purchased them straight from the store. Because the remaining poison in the bottle was poured down in the space, it's impossible to determine how much she consumed. Due to a lack of certain types of equipment, the student clinic promptly and without any aid moved her to a tertiary hospital nearby.

She arrived at the hospital with a two-hour history of nausea and sporadic vomiting as well as an hour of persistent vomiting, increased salivation through the mouth, chills, progressive sweating, breathing difficulties, and dizziness. Clinical evidence of her susceptibility to the poison, clinical features, and the presence of a garlic-like odor frequently round out the diagnosis. Gastric lavage was done right away after she was admitted without any vital signs, laboratory testing, or physical examination. On admission, she had a mean arterial blood pressure of 88/59 mmHg, a pulse rate of 102 beats per minute, a body temperature of 35.9 °C, a height of 1.59 m, a weight of 58 kg, an oxygen saturation level of 89% in room air, and a respiratory rate of 22 breaths per minute (Table 1).

**Table 1:** Vital signs of three consecutive days from admission to discharge of the patient

Days	Typical vital signs			
	Blood pressure (mmHg)	Heart rate (bpm)	Respiratory rate (bpm)	Saturated oxygen (%)
Day 1	88/59	102	22	89
Day 2	115/86	77	14	98
Day 3	125/83	71	15	96

At arrival time, laboratory investigations of the patient revealed blood urea nitrogen of 24 mg/dl (normal value: 6-21 mg/dl), serum creatinine of 1.4 mg/dl (normal value: 0.59-1.04 mg/dl), serum bicarbonate level of 20.0 mEq/L (normal value: 22-29 mEq/L), serum phosphate level of 2.9 mg/dl (normal value: 2.8-4.5 mg/dL), white blood cell count of 13200 cells/mm3 (normal value: 4500-11000 cells/mm3), serum sodium level of 129 mEq/L (normal value: 135-145 mEq/L), and serum potassium level of 3.6 mEq/L (normal value: 3.5-5.5 mEq/L) (Table 2).

**Table 2:** Laboratory investigations of three consecutive days from admission to discharge of the patient

Parameter	June 22/2022	June 23/2022	June 24/2022
Blood urea nitrogen (mg/dl)	24	21	19
Serum creatinine (mg/dl)	1.4	1.85	1.3
Serum bicarbonate level (mEq/L)	20.0	25	29
Serum phosphate level (mg/dl)	2.9	2.7	3.8
White blood cell count (cells/mm^3^)	13200	11200	10950
Serum sodium level (mEq/L)	129	134	146
Serum potassium level (mEq/L)	3.6	3.9	5.3

Her Glasgow Coma Scale reading was 9/15 upon admission, according to the emergency department's neurological evaluation. During a physical examination, it was discovered that she had heavy sweating, saliva dripping from her mouth, and bilateral vesicular breath noises in her chest. Her stomach was smooth with normal bowel sounds on auscultation, but her pupils were constricted in both directions, she was sleepy with dyspnea, and there was noticeable discomfort near the epigastrium.

She was intubated with mechanical ventilation eight hours later for hypotension and profound dyspnoea. She was started on two liters of intranasal oxygen per minute upon admission via a nasal cannula. She received atropine 0.15 mg intravenously on the same day, with the dose being doubled every 10 minutes to achieve atropinization, and then a bolus dose of 500ml of 0.9% normal saline was started right away. Within three days, she had recovered and had been sent back to her university.

## Discussion

For farming and home usage, organophosphate pesticides are widely-used and often obtainable over-the-counter insecticides in Ethiopia, and they are accountable for the most extensive number of deaths following ingestion while trying to commit suicide [9]. Unusual cardiac toxicity from organophosphate poisoning includes myocarditis and pericarditis with interstitial inflammation [10]. Following organophosphate exposure, heart toxicity goes through these three phases: (i) A brief course or the initial phase is characterized by mild sympathetic action, which normally lasts for a few minutes and presents as hypertension and elevated breath pressure [11]. (ii) A second phase is subsequently followed by an additional extended period of excessive cholinergic toxicity, which may cause life-threatening beat irregularities, ST-T shifts, reduced lung capacity, and hypotension. (iii) The third stage, known as the "prolonged phase," is often associated with a long QT interval and polymorphic VT and may result in an early death [12].

When compared to the three stages of organophosphate exposure to heart toxicity, the patient, in this case, had an elevated pulse rate (the first phase) and hypotension on the day of arrival (the second phase). She arrived at the hospital on time and received treatment immediately, so she didn't undergo the "prolonged phase of cardiac poisoning."

Organophosphate poisoning can result in the following three major syndromes: i) Acute cholinergic syndrome: Organophosphate poisoning symptoms often appear one to two hours after exposure. ii) Intermediate syndrome: This condition usually appears one to four days after exposure to organophosphate poisoning, but it can even appear a week later. The main symptom of Intermediate syndrome is neck weakness, which develops into respiratory paralysis and death. iii) Organophosphate poisoning induced delayed polyneuropathy: A rare delayed consequence of acute organophosphate poisoning is delayed polyneuropathy. Organophosphate poisoning is delayed neuropathy brought on by organophosphates. After 10 to 21 days of exposure, a distal ascending neuropathy known as delayed toxicity develops [13].

The patient in this case exhibited acute cholinergic syndrome and intermediate syndrome, two of the three primary organophosphate poisoning symptoms. Because she recovered from pesticide organophosphate poisoning within three days of being admitted, she did not experience delayed polyneuropathy due to organophosphate poisoning. Organophosphate poisoning has several well-known clinical symptoms, including bradycardia, miosis, bronchospasm, increased salivation, lacrimation, urine, muscular paralysis, diarrhea, and increased salivation [14]. Organophosphate poisoning causes central respiratory depression, agitation, convulsions, and coma in the central nervous system (CNS), which is home to nicotinic and muscarinic receptors [15]. Organophosphate poisoning may show symptoms immediately, such as respiratory distress or an intermediate syndrome, or it may take time to show symptoms [9,16]. In this case report, the patient had been experiencing nausea and vomiting for two hours as well as chronic vomiting for one day, increased salivation through the mouth, chills, progressive sweating, difficulty breathing, and dizziness. Gastric lavage was done right away after she was admitted without any vital signs, laboratory testing, or physical examination. Organophosphate poisoning is treated by resuscitating the patient, administering oxygen through a nasal cannula, the muscarinic antagonist, especially atropine sulfate, fluids to balance electrolytes, and an oxime to reactivate acetylcholinesterase by removing the phosphate group [17,18].

Limitations

Due to the electrocardiography machine being old and inoperable, there were no results when this case report was being completed. She ingested an unknown amount, making it impossible to assess the severity and complexity of her situation at arrival time.

## Conclusions

Acetylcholinesterase is rendered inactive by the phosphorylation of the active site by organophosphate poisoning chemicals, which also results in an excess of nicotinic and muscarinic receptors. Organophosphate poisoning typically causes symptoms like nausea, vomiting, diarrhea, urine incontinence, blurred vision, salivation, lacrimation, bronchorrhea, bradycardia, hypotension, muscle paralysis, fasciculation, disorientation, seizures, coma, and respiratory failure. The best medicine for treating organophosphate poisoning is atropine sulfate because it prevents acetylcholine from acting at muscarinic cholinergic synapses after organophosphate poisoning inhibits acetylcholinesterase.

To reduce the frequency of consumers being poisoned, the Ethiopian government ought to have placed restrictions on the vendors who were permitted to sell the poisoned goods. Manufacturers, regulatory agencies, and national poison control centers should be required to participate in the minimization of the rise in intentional poison ingestion among adults. Governments should spread awareness of preventative measures, including redesigning containers with warning labels and primary poison prevention methods. The author urges that all people should cope with the problems they face by solving them peacefully rather than committing suicide.
